# The metabolic, microbial and immunological demands **of** pneumococcal colonisation

**DOI:** 10.1371/journal.ppat.1013675

**Published:** 2025-11-13

**Authors:** Daniel R. Neill, Thomas B. Clarke

**Affiliations:** 1 Division of Molecular Microbiology, University of Dundee, Dundee, United Kingdom; 2 Department of Infectious Disease, Centre for Bacterial Resistance Biology, Imperial College London, London, United Kingdom; Carnegie Mellon University, UNITED STATES OF AMERICA

## Abstract

*Streptococcus pneumoniae* (the pneumococcus) causes a range of life-threatening diseases including pneumonia, sepsis and meningitis. Despite widespread vaccine deployment, pneumococcal disease remains a leading cause of global mortality. The human nasopharynx is its principal ecological niche, and all pneumococcal disease originates from this reservoir of organisms. Acute infections are, however, an evolutionary dead-end for the pneumococcus. What sustains the pneumococcus within human populations are cycles of colonisation and transmission. To persist within the nasopharynx, it must overcome the physical and immunological barriers established by the host while acquiring sufficient nutrients to proliferate in the face of competing airway microbes. Here, we outline the metabolic, microbial, and immunological challenges of colonisation, and the often-competing demands of transmission, which together have shaped the pneumococcus into one of the most formidable human pathogens.

## Host defences against pneumococcal colonisation

**Fig 1:**
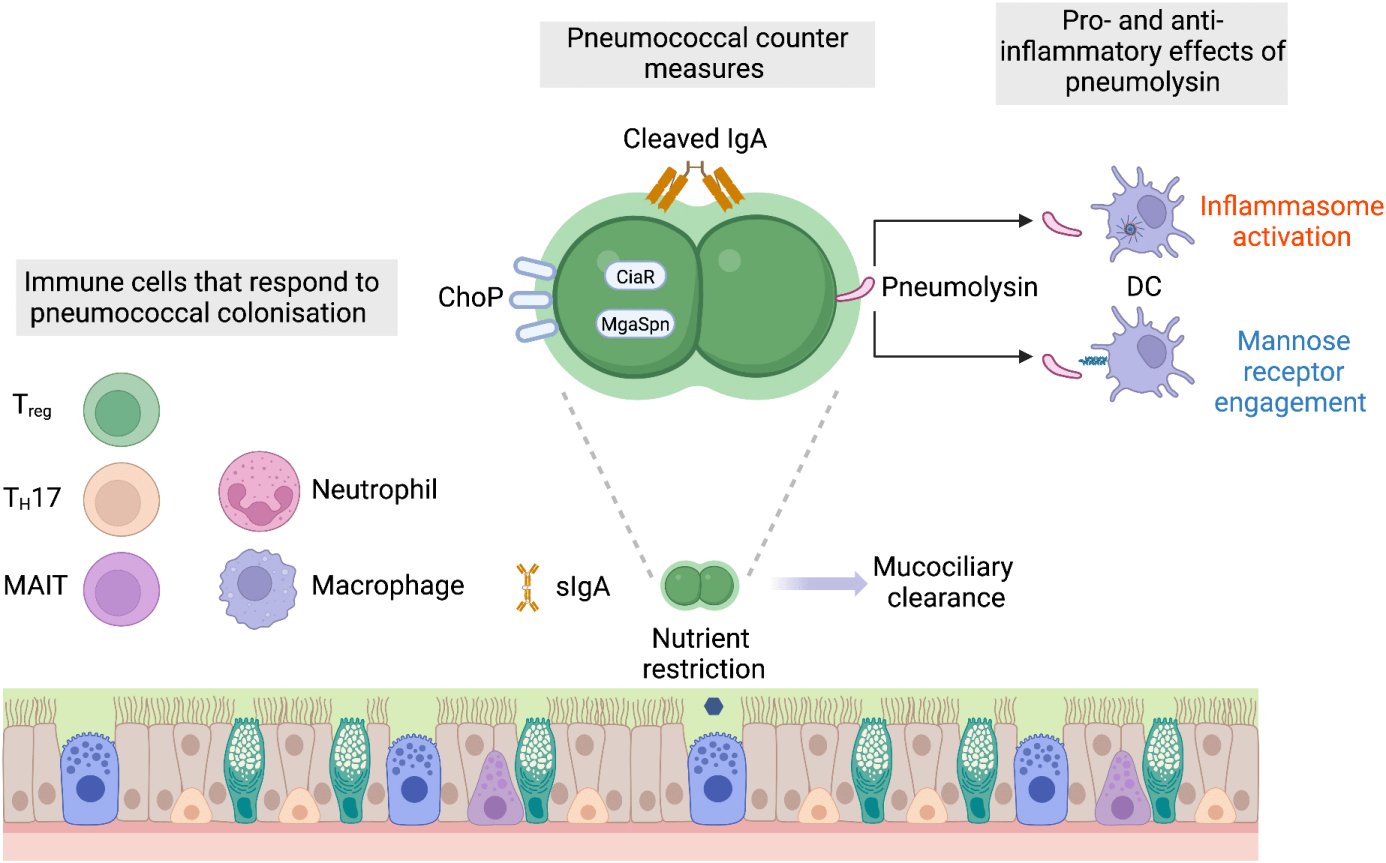
Host defences against pneumococcal colonisation. The host restricts pneumococcal colonisation through nutrient limitation and goblet cell secretion of mucus. Mucus-entrapped bacteria are cleared via the mucociliary escalator. Secretory IgA neutralises pneumococcal effectors and limits epithelial adherence. Recognition of pathogen-associated molecular patterns by TLRs and NLRs activates inflammatory cascades, recruiting IL-17–producing CD4⁺ T cells that coordinate neutrophil and macrophage responses. MAIT cell-derived IFN-γ and TNF have been proposed to also contribute to colonisation control. Pneumococcal countermeasures: The negatively charged capsule reduces mucus entrapment, while phase variation alters capsule thickness. Glycosyl hydrolases degrade mucin glycans, aiding mucus escape. IgA protease cleaves IgA, thus promoting evasion of antibody-mediated immunity. Fab binding can mask capsule charge, enhancing epithelial adherence. ChoP, regulated by MgaSpn and CiaR, facilitates epithelial adherence via PAFr. Pneumolysin has both pro- and anti-inflammatory effects. After host cell membrane engagement, pneumolysin triggers inflammation via NLRP3. It also modulates immunity through CD206 engagement on dendritic cells and macrophages. These responses promote T regulatory cell activation, prolonging nasopharyngeal carriage. Created in BioRender. Clarke, T. (2025) https://BioRender.com/cjflzs3.

Pneumococcal carriage prevalence is highest early in life, between 2and 3 years of age, and in low to middle-income countries, where it ranges from 26.7% to 90.7% [1]. Carriage declines with age and rates are generally less than 10% in adulthood. In infants, the duration of nasopharyngeal colonisation is approximately 60 days, whereas in adults it is approximately 31 days, although these timespans can vary considerably between individuals [2–5]. Recognition of colonising pneumococci in the nasopharynx engages several conserved host defence mechanisms, which have been examined using animal models and human challenge (Fig 1). Broadly, these begin by preventing pneumococcal attachment to the airway and restricting nutrient availability to limit the opportunities for growth for any pneumococci that have attached. These preventative measures are complemented by innate and adaptive immune responses aimed at destroying the pneumococcus. The pneumococcus must therefore have mechanisms to contend with and surpass these defences if it is to successfully establish colonisation.

One of the most important physical barriers that the pneumococcus must overcome is mucus secreted by goblet cells and submucosal glands in the airway [6]. Mucus is composed of extensively glycosylated and sulphated mucin proteins and water, forming a negatively charged gel overlaying the epithelium. Pneumococci that become entangled in mucus are conveyed by the beating of cilia on the underlying epithelium to the oropharynx where they are swallowed and cleared. These processes help restrict access to the epithelium, preventing nasopharyngeal attachment [7]. One of the most important factors that enables the pneumococcus to contend with mucus is its capsular polysaccharide. There are greater than 100 structurally different capsular types known [8]. Capsular polysaccharides are important in many aspects of pneumococcal biology, including colonisation and environmental survival during transmission, as well as being the most important target of pneumococcal vaccines [9]. Most capsule types have an overall negative charge, and this helps disentangle the pneumococcus from airway mucus, allowing it to reach the airway epithelium [10]. Evasion of mucus entrapment is also facilitated by the actions of glycosyl hydrolases that sequentially remove sugars from mucus proteins [11,12]. Avoiding mucus entrapment allows for intimate association with the epithelium, increasing the duration of colonisation, with bacteria later shedding back into the mucus layer to ensure onward transmission, reviewed in [13]. Capsule production can vary, with the pneumococcus undergoing spontaneous phase variation, switching between opaque variants that produce higher amounts of capsule and transparent variants that produce less of this important surface polymer. Phase variation is controlled by a random six-phase genetic switch, with the apparent dominance of opaque variants in invasive disease and transparent variants in carriage presumably the result of selection within host [14–16]. The benefit of phase variation in the early part of colonisation is that the transparent variants attach to, and subsequently invade, epithelial cells more effectively [17–19]. This likely stems from exposure of proteins, such as PavB, PsaA and PspC, associated with attachment to the epithelium [20,21].

A further barrier to the pneumococcus in the airway is provided by secretory IgA (sIgA). This is the most abundant antibody isotype produced at mucosal surfaces and protects against bacterial colonisation through multiple mechanisms. Studies on a range of bacterial pathogens have revealed that these include blocking attachment to epithelial receptors, promoting agglutination and clearance, and restricting the activity of bacterial virulence factors. In the case of pneumococcal colonisation, sIgA protects through its ability to promote agglutination via binding to the Pilus-1, enhancing clearance of the pneumococcus from the airway [22]. To counter the effects of sIgA, the pneumococcus, like other opportunistic airway pathogens including *Haemophilus influenzae* and *Neisseria meningitidis*, produces an IgA1 protease. Up to 90% of the sIgA in the airway is IgA1. The activity of the IgA1 protease cleaves IgA recognising the pneumococcal capsule, liberating the Fc portion of IgA and generating Fab fragments that retain the ability to bind to the capsule, enhancing the attachment of the bacterium to epithelium [22]. Mechanistically, this abrogates the repulsive effect of the negative surface charge on the capsular polysaccharide and facilitates pneumococcal binding to the airway epithelium. Airway epithelial attachment is further enhanced by the binding of host platelet-activating factor receptor (PAFr) through bacterial surface phosphorylcholine (ChoP) [23]. Host PAFr expression is upregulated during inflammation, including that stimulated by viral infection or inhaled pollutants [24,25]. Pneumococcal ChoP production is under tight environmental control, via the global transcriptional regulator MgaSpn (MgrA) and the CiaRH two-component regulatory system. CiaRH is also environmentally regulated, with *ciaRH* mRNA under the control of an RNA thermosensory element that triggers increased translation under elevated temperatures associated with fever [26]. At the protein level, CiaRH senses and responses to airway sialic acids via a mechanism that bypasses that histidine kinase [27]. Thus, ChoP production is tightly linked to niche conditions, suggesting a critical role in host–pathogen interactions.

Pneumococci that manage to reach the epithelial surface are recognised by the innate immune system, activating an inflammatory programme to control colonisation. This is a concerted response by the airway epithelium and macrophages and neutrophils recruited to the nasopharynx. Pattern recognition receptors, particularly the Nod-like receptors (NLRs) and Toll-like receptors (TLRs) are critical for this surveillance [28,29]. The importance of TLRs is highlighted by clinical data from people with mutations in the genes encoding MYD88 or IRAK4, adaptor proteins required by most TLRs. Individuals with these mutations suffer from recurrent infections by a limited repertoire of pathogens, with the most common disease being invasive pneumococcal infection [30,31]. Recognition of the pneumococcal surface glycoconjugate polymers, including peptidoglycan and teichoic acids, by these pattern recognition receptors engages a range of signalling pathways such as the mitogen-activated kinase and transforming growth factor-beta signalling in the epithelium [32–34]. Activation of these pathways opens the epithelial barrier, allowing for antimicrobial factors to enter the luminal spaces where pneumococci are located. One of the first antimicrobial effectors recruited to the airway lumen are neutrophils [35]. During the host’s first interaction with the pneumococcus, however, recruited neutrophils are unable to clear colonisation. Rather, expulsion requires Tlr2 activation and IL-17A and CD4+ T cells to recruit macrophages, which have the requisite level of antimicrobial activity to effect pneumococcal clearance [35]. During secondary clearance, IL-17A and memory CD4+ T cells promote a more robust influx of myeloid cells into the nasopharynx, which more rapidly clear colonisation. In contrast to primary colonisation, neutrophils are crucial in this secondary response. In addition to TLRs, the activation of the NLR, Nod2 also orchestrates recruitment of macrophages into the upper airway. Nod2 activation by the pneumococcus stimulates Ccl2 production and macrophage recruitment, complementing TLR-mediated defence [36].

Detailed dissection of the mechanisms of pneumococcal clearance from the nasopharynx has relied heavily on animal models that closely recapitulate human colonisation. Emerging data from humans indicates that the immunological effector mechanisms important for pneumococcal clearance may be more complex that we currently appreciate. While there are data derived from human studies that support the development of Th17 cell responses in the lymphoid tissues associated with the nasopharynx, namely the nasal-associated lymphoid tissue [37–39], other experimental human carriage data have not found any association of pneumococcal colonisation with any CD4+ T memory cells, including Th17 cells [40]. It is important to note, however, that data from these human carriage studies is limited in duration to 10 days and that animal studies indicated that the major role for these cells may be most marked after that time. What these human carriage studies have revealed is the potential importance of mucosal-associated invariant T (MAIT) cells, a class of unconventional T lymphocytes with innate cell-like effector functions that recognise a restricted antigen repertoire presented by the MHC class I-like protein MR1 [41,42]. In people where the pneumococcus was unable to establish colonisation, MAIT cells responded more vigorously to stimulation with the pneumococcus, producing higher levels of TNF and IFNγ, compared to MAIT cells from people where the pneumococcus was able to establish colonisation. Collectively, multiple studies have established that the inflammatory response in the airway is central to protecting against colonisation. The potential toxicity of inflammation, however, means the host rarely lets it operate unchecked, and the inflammatory response to pneumococcal colonisation is no exception. The major immune cell population responsible for moderating the effects of inflammation are T regulatory cells (Tregs) and they play an important role in the immune response to colonisation. In children colonised by the pneumococcus, nasal-associated lymphoid tissues contain a higher proportion of CD4+ T cells phenotypically defined as Tregs, as compared to uncolonized children [39]. These lymphocytes dynamically regulate the immune response within the nasopharynx during colonisation. If the colonisation density of the pneumococcus is low, the activity of Tregs subdues the inflammatory response, limiting inflammatory clearance of colonisation. If colonisation density is higher, the role of Tregs diminishes and the inflammatory response predominates, promoting clearance of colonisation [43]. This suggests that the host carefully monitors the levels of pneumococcal colonisation to respond optimally. During low levels of colonisation, the host tolerates the pneumococcus, as the potential cost of deploying an inflammatory response is not merited. As pneumococcal levels increase, the risks of the inflammatory response become worth taking to limit the dangers associated with higher pneumococcal burden.

Rather than trying to temper this inflammatory milieu in the upper airway, the pneumococcus has adapted to embrace it. The pneumococcus produces a cholesterol-binding pore-forming toxin, pneumolysin, that is a potent activator of inflammatory cascades [44]. Pneumolysin is considered one of the major virulence factors produced by the pneumococcus and is conserved in all serotypes, underscoring its importance for pneumococcal biology. In general, pneumolysin binds to cholesterol in the cell membrane causing membrane disruption, which is followed by disruption of osmotic homeostasis and then cell death. Because of this direct damage to the plasma membrane, the activity of pneumolysin is detected by the NLRP3 inflammasome [45]. This behaviour is highly conserved, although the sequence type (ST)306 lineage of serotype 1 pneumococcus and the ST53 lineage of serotype 8 produce variants of the protein that lack haemolytic activity, which likely contributes to the atypical patterns of disease caused by these sequence types [46]. Intriguingly, pneumolysin can also promote immunoregulatory host responses, via its effects on dendritic cells and macrophages, where it binds the mannose receptor (CD206) [47]. This interaction suppresses proinflammatory cytokine production via SOCS1 and promotes internalisation of pneumococci into non-lysosomal compartments. Dendritic cells stimulated via pneumolysin-CD206 polarise T cells towards a Foxp3-expressing immunoregulatory phenotype. The immunomodulatory activity of pneumolysin in this context likely helps to balance the potent inflammatory effects that derive from toxin-driven host cell lysis. Counterintuitively, given its broad conservation across serotypes, the activity of pneumolysin is associated with faster clearance of the pneumococcus from the nasopharynx [48]. This apparent detrimental role during colonisation, is however, offset by the advantage it provides by stimulating the inflammation required to promote respiratory secretions to propel onward transmission [49]. Thus, the pneumococcus exploits inflammation to spread between hosts but also regulates inflammatory signals to ensure longevity of colonisation within a host.

## Nutritional and metabolic demands of colonisation

Survival within the nasopharynx requires not only avoiding killing but also scavenging sufficient nutrients for proliferation. The pneumococcus uses fermentation via the Embden–Meyerhof–Parnas glycolysis pathway to generate energy [50]. Pneumococcus has no Entner–Doudoroff pathway, nor does it have an electron transport chain or the full suite of the enzymes required for the tricarboxylic acid cycle [51]. In addition to these limitations in catabolic metabolism, pneumococci exhibit a restricted capacity for amino acid biosynthesis and are unable to generate a complete set of amino acids *de novo* [50–52]. Thus, acquiring sugars and amino acids within the nasopharynx are central objectives for the pneumococcus. Much of the pneumococcal genome therefore encodes for enzymes that degrade complex glycans and proteins and transport systems that facilitate the uptake of the simple sugars and amino acids they produce. This enables metabolic flexibility in the face of the nutrient-poor conditions found in the nasopharynx. The pneumococcus can grow on over 30 carbohydrates and has more than 40 known or predicted enzymes that have the potential to cleave glycosidic bonds. This glycan-degrading machinery can catabolise a wide range of host *N*-linked and *O*-linked glycans and even glycans derived from plants that are common components of our diet. More than 30 genes encoding carbohydrate transporters have been identified in the pneumococcal genome, including ATP-binding cassette (ABC) transporters and phosphoenolpyruvate-dependent phosphotransferase systems, reviewed in [53]. The substrate specificities of these systems are now being defined, revealing considerable redundancy.

## Flexible metabolism in colonisation and disease

Different metabolic strategies are required by pneumococcus during colonisation, transmission and for survival in host niches beyond the nasopharynx [54,55]. In an experimental evolution study in mouse colonisation and disease models, metabolic genes were found to be under selection in both nasopharynx and lungs. Whilst metabolic loci accounted for around 40% of identified non-synonymous mutations, selection operated on different genes in the upper compared to the lower airways, suggesting differing metabolic strategies are optimum in different respiratory compartments [56].

**Fig 2:**
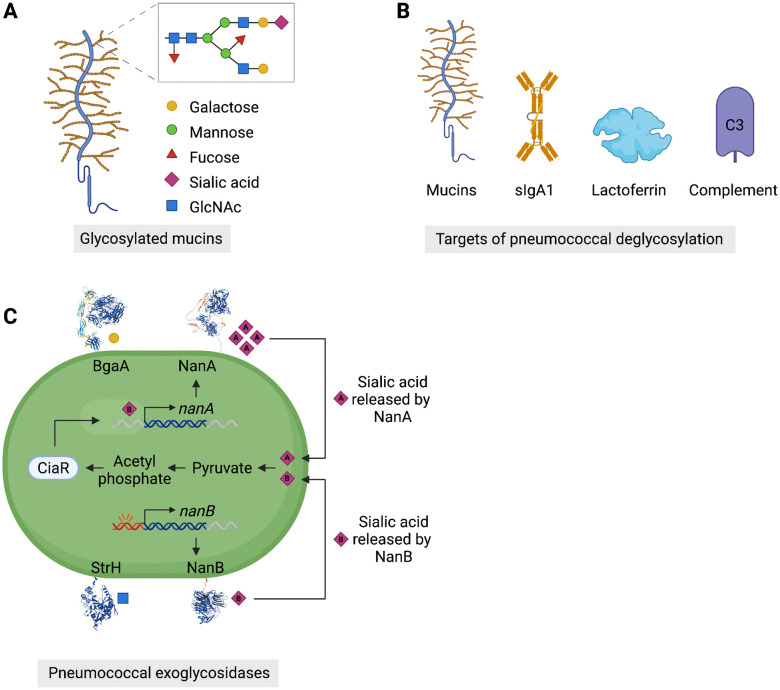
Regulation of pneumococcal glycosyl hydrolases and host glycan utilisation during colonisation. **(A)** Characteristic structure of glycosylated mucins, terminating in sugars that are substrates for the three principal families of pneumococcal glycosyl hydrolases. **(B)** Host targets for pneumococcus-driven deglycosylation. Enzyme activity yields free sugars for growth, evasion of mucus entrapment, exposure of binding sites on the epithelium and inactivation of host immune defence proteins. **(C)** Regulation of neuraminidase activity involves a complex host-sensing network. The propensity of the *nanB* promoter to acquire mutations allows colonised pneumococci to optimise transcriptional activity at the locus to local conditions during single infections. In the presence of host glycans, NanB activity yields free sialic acids for import, where they both derepress the *nanA* locus and are fed into pyruvate metabolism, leading to production of acetyl phosphate that can act as a phosphor donor for the CiaR response regulator, which can further upregulate NanA. CiaR production is regulated by an RNA thermosensory element, providing another means of modulating neuraminidase activity in response to changing host environmental conditions. Created in BioRender. Clarke, T. (2025) https://BioRender.com/m93hdsd.

Carbon catabolite repression suppresses alternative metabolism under conditions where glucose is available. Disease sites, including lungs and the bloodstream, are relatively glucose-rich, but during acute infection, following a brief surge in availability [57], the host restricts glucose to limit bacterial growth and to fuel the activities of metabolically demanding immune cells, such as neutrophils. Thus, access to alternative sugar sources is key to pneumococcal success within the host and this is especially true in nasopharynx, where glucose availability is limited even in the absence of inflammation [58]. Alternative sugars are not freely available but are sequestered within glycan structures that decorate airway mucins (Fig 2). Pneumococcus sequentially removes these through the actions of sialidases (NanA, B and C), galactosidases (BgaA) and *N*-acetylglucosaminidases (StrH), before their uptake via dedicated transporter systems.

Sialic acids often form the terminal residues on host airway glycoproteins. Pneumococcus produces three neuraminidases: NanA, NanB and NanC. NanA is present in all strains and has been extensively characterised, revealing roles in biofilm formation, immune evasion and invasion of epithelial cells, in addition to its principal function in cleavage of α2–3 and α2–6-linked sialic acid residues from glycans [59–61]. NanB is present in more than 95% of pneumococcal strains and NanC in almost 50% of isolates [62]. Neuraminidase expression is under strict environmental control, with NanA, which provides the bulk of the enzymatic activity, relying upon the prior liberation of free sialic acids by NanB to derepress its expression [11]. In this way, the pneumococcus ties production and activity of sugar acquisition machinery to the availability of those sugars within the host. A role for host sensing in pneumococcal sialic acid acquisition is further suggested by a study demonstrating differing infection outcomes in mice expressing a human-like sialic acid profile (with terminal Neu5Ac), as compared to those with the typical mouse airway glycan structure that terminates with Neu5Gc. The human sialic acid profile stimulated upregulation of NanA, leading to elevated bacterial loads during infection. This process was under the control of the CiaRH two-component regulatory system, following uptake of sialic acids by the SatABC transporter [27]. Whilst NanA expression is tightly regulated, pneumococcus has an inherent propensity to vary expression of NanB. The *nanB* promoter contains an 8–13 base pair poly-adenine nucleotide signature that dramatically increases the likelihood of strand slippage during DNA replication, leading to a high mutation rate at the locus, altering the level of transcription of *nanB* operon genes (Fig 2). Within single nasopharyngeal carriage episodes in humans, two or more *nanB* promoter sequences are frequently identified and experiments in mice demonstrate that different *nanB* promoter sequences are associated with different colonisation and disease phenotypes. Mixed infections with otherwise isogenic strains containing differing promoter sequences showed improved colonisation potential compared to single-strain infections [12]. The readily mutable poly-adenine promoter signature at the *nanB* locus might provide an evolutionary strategy to explore fitness landscapes within host, by varying the transcriptional profile of host-interfacing sugar acquisition machinery. Between 1% and 3% of transcriptional units in the pneumococcal genome contain 8 or more consecutive adenine residues within 100 base pairs of the transcriptional start site, suggesting this strategy might be of broader relevance, but whether this is a specific feature of metabolic loci remains to be determined.

The β-galactosidase, BgaA, plays a similarly critical role at the host-pathogen interface. In addition to its role in galactose acquisition, BgaA mediates attachment to host via interaction of its carbohydrate-binding modules with host surface lactose or *N*-acetyllactosamine [63]. Furthermore, BgaA stimulates immune clearance via recognition of its conserved degron motif by the host ubiquitylation machinery. Ubiquitylation of BgaA targets intracellular pneumococci for proteasomal degradation. Whilst principally an extracellular pathogen, there is a growing body of literature suggesting that intracellular survival contributes to pneumococcal success during both colonisation and disease, reviewed in [64]. Alongside the functions described above, the neuraminidases, BgaA and StrH act in concert to modify or limit the activities of glycosylated host immune proteins, including complement proteins, lactoferrin and sIgA [61,63,65] (Fig 2). These enzymes have multifaceted roles in colonisation and disease, acting as host-sensors, providers of vital metabolic resources, and interfacing with the host immune system. However, their contributions to infection processes are strongly context dependent, as revealed by the phenotypes of gene deletion strain lacking one or more of the enzymes. Such strains show clear fitness defects in some disease contexts but not in others, and colonisation potential is often only subtly altered relative to wild-type [65–67], suggesting a level of metabolic redundancy that might be expected for a niche-specialist like pneumococcus. A remaining unknown for this field of research is the extent to which glycan or free sugar bioavailability in the airway varies between individuals, throughout the airway environment and over the course of infection. The rapidly developing field of metabolomics offers opportunity to shed light on airway metabolic landscapes and pneumococcal human challenge models provide a means of investigating how pneumococcal colonisation perturbs metabolic equilibrium.

Different pneumococcal lineages have varying metabolic preferences and sugar utilisation capacities. The reference strain D39 lacks a functional neuraminate lyase, preventing it from using sialic acids for growth, but it retains the capacity to process sialic acid-containing glycans [68]. A study of serotype- and sequence type-matched blood and ear isolates found reduced raffinose metabolism in the latter due to a single nucleotide polymorphism in the *rafR* transcriptional regulator [69]. This SNP affected tropism for different disease niches, with the raffinose-utilising blood isolate achieving higher levels in the lungs compared to the ear. Introducing the *rafR* SNP to the blood isolate reversed this tropism. Galactose utilisation via the tagatose and Leloir pathways also affects virulence, with the relative contribution of the two catabolic pathways determined by the physical and chemical environment during infection. Both pathways are repressed by glucose and sialic acids and the tagatose pathway is inducible at lower galactose concentrations than those required to activate the Leloir pathway [55]. Pneumococcal mutants expressing a non-phosphorylatable form of the GalR transcriptional activator of the Leloir pathway have a reduced capacity to colonise nasopharynx, ear, lung, and blood [70]. Changes in carbon source utilisation can have pleiotropic effects on pneumococcal gene expression and influence virulence factor expression, including the capsule. Pneumococci grown on mannose or *N*-acetylglucosamine have reduced capsule thickness relative to those grown on glucose or galactose, whilst growth on mannose improves epithelial adherence [71]. The effects of glucose on capsule biosynthesis are mediated through binding of catabolite control protein A to the promoter of the *cps* gene cluster [72]. The effects of carbon source on capsule thickness are strain and serotype-dependent, and for some lineages, including those of serotype 6F, carbon source also influences capsule structure, with a relative increase in the proportion of glucose in the capsule when it is the sole carbon source available for growth [73,74].

The link between pneumococcal carbon utilisation and colonisation is well established, but how transmission is affected by metabolism is only just beginning to be revealed. A high-throughput screen using a pneumococcal transposon library in a ferret transmission model identified 87 genetic elements required for transmission, of which more than 30% had a role in metabolism, with carbohydrate metabolism most prominent [75]. Follow-up studies to define the contributions metabolic gene products make to the microbial physiology that drives transmission should provide insights into how pneumococcus balances the sometimes competing demands of colonisation and onward transmission.

## Beyond carbohydrate metabolism

Carbohydrate processing, uptake and metabolism have been the focus of research because of their centrality to pneumococcal success in the airways. Other branches of metabolism, however, also play important roles in coordinating pneumococcal colonisation. Branched-chain amino acids (BCAAs) represent a key metabolic node via their effects on the master nutritional regulator, CodY, and the bacterial stringent response. BCAAs, especially isoleucine, bind directly to CodY, enhancing its affinity for DNA and leading to suppression of genes of the CodY regulon, many of which are dispensable in times of relative nutrient abundance [76]. The stringent response of pneumococcus is only partially characterised, but the species encodes at least one RelA/SpoT homolog, Rel_Spn_, capable of both synthesising and hydrolysing the nucleotide alarmone (p)ppGpp [77]. As in other species, BCAA limitation is a trigger of the pneumococcal stringent response, activating a regulatory program to arrest growth and promote alternative carbon metabolism, in part via links with the carbon catabolite repression system. Another study identified a link between misaminoacylated tRNA accumulation and stringent response activation, shutting down translation to prevent toxic accumulation of mistranslated or misfolded proteins [78]. For pneumococcus, the stringent response likely contributes to the ability to survive periods of nutrient limitation or environmental perturbation. Of note, the regulon under Rel_Spn_ control has been reported to include pneumolysin, suggesting potential for the stringent response to have consequential effects on colonisation [77]. During homeostasis there is limited leucine, isoleucine and valine available in the nasopharynx, but during pneumococcal carriage, BCAA levels increase. Strains that are better adapted to colonisation have an enhanced ability to boost nasopharyngeal BCAA availability [58]. The extent to which airway BCAA limitation drives CodY or stringent response-mediated transcriptional repression is unclear, although mutations in Rel_Spn_ have been shown to affect resistance to neutrophil killing and competitiveness for colonisation of the mouse nasopharynx [79]. The same study reported that SNPs in a second RelA/SpoT homolog had similar effects. This putative RSH protein has not been characterised but appears to lack the hydrolase domain present in Rel_Spn_, suggesting it may act as an additional synthase.

## Microbial competition and cooperation within the airway

**Fig 3:**
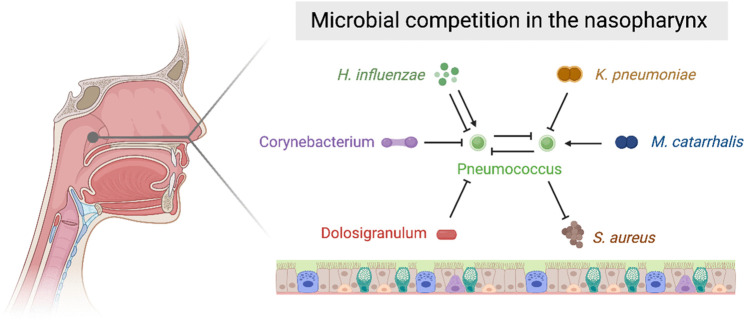
Microbial competition shaping pneumococcal ecology in the nasopharynx. The pneumococcus is part of a complex network of ecological interactions that influence its growth and survival within the nasopharynx. Pointed arrows indicate a positive relationship promoting higher levels of the target organism. For example, *Moraxella catarrhalis* promotes pneumococcus growth. By contrast, blunt-ended arrows indicate an inhibitory effect on target organisms. For example, *Corynebacterium* and *Dolosigranulum* inhibit pneumococcus, while *Klebsiella pneumoniae* also suppresses its growth. Created in BioRender. Clarke, T. (2025) https://BioRender.com/ewryf7x.

To establish colonisation, the pneumococcus must successfully navigate the complex microbial landscape of the upper airway. Within its nasopharyngeal niche, each strain engages in a diverse array of ecological interactions—competing not only with other pneumococcal strains and potentially pathogenic bacterial species, but also with benign commensal organisms (Fig 3). Understanding the ecological rules of these bacterial inhabitants is critical to fully understand what determines the outcome of pneumococcal colonisation.

The nasopharyngeal microbiota of young children is characterised by a lower diversity but higher microbial load than that of adults [80]. Recent sequencing studies have revealed that nasopharyngeal microbiota in the first few weeks of life is often dominated by Staphylococcus, which is then outcompeted by Corynebacterium and Dolosigranulum [80–82]. In comparison to the gut, which stabilises in childhood after 2–5 years, the nasopharyngeal microbiota stabilises between adolescence and adulthood. In adulthood, the nasopharynx is composed of approximately 15 core genera, which include Staphylococcus, Prevotella, Rothia, Moraxella, Corynebacterium, Dolosigranulum and Streptococci [80]. By contrast, the oropharynx is characterised by higher bacterial load and has 22 core genera, including Veillonella, Prevotella, Streptococci and Leptotrichia [80]. A negative association between pneumococcal colonisation and either Corynebacterium or Dolosigranulum has been documented in multiple surveys of the upper airway microbiome [80,83]. These observations are complemented by experimental data which has demonstrated that the combined activity of *Dolosigranulum pigrum* and *Corynebacterium pseudodiphtheriticum* can inhibit pneumococcal growth [84,85]. The large number of poorly, or completely uncharacterised microbes in the airway means that the pneumococcus is likely involved in many interactions of which we are currently unaware. The impact of the microbiota on the pneumococcus extends beyond the local interactions within the upper airway. The gut microbiota enhances resistance to the major forms of acute pneumococcal disease: pneumonia, sepsis and meningitis. This occurs via the dissemination of immunologically active cell wall glycoconjugates from the gut which prime the antimicrobial activity of tissue resident macrophages in organs throughout the host and neutrophils in the bone marrow [86–88]. This organism-wide priming of innate cell antimicrobial function allows the host to effectively clear invading pneumococci from the lung, bloodstream, spleen and brain. Thus, the gut microbiota is also a decisive factor in keeping the pneumococcus restricted to the nasopharynx. Greater mechanistic insight into the microbial interactions occurring within the upper airway and with microbes at other mucosal sites could reveal novel strategies to block pneumococcal colonisation.

Complementing these recent microbiome studies, there is a rich history of mechanistic investigation interrogating intraspecies and interspecies competition within the nasopharynx. Multiple pneumococcal strains can simultaneously colonise the nasopharynx. A recent human carriage cohort study determined that presence of a within-host pneumococcal competitor was associated with a 33% increased chance of clearance and a 54% reduction in carriage establishment for the invading strain. Interestingly, in the absence of antimicrobial treatment, drug-susceptible lineages appeared to be at a competitive advantage, suggesting antimicrobial resistance involves fitness trade-offs [89]. Serotype replacement—where a pneumococcal strain not covered by a vaccine replaces a strain eliminated by vaccination—further highlights that there is intraspecies competition between pneumococci for residence in the nasopharynx. Competition between pneumococcal strains is thought primarily to be mediated via bacteriocins encoded by the conserved bacteriocin-like peptide (*blp*) locus [90]. Bacteriocins are small proteins that damage cell surface structures like the plasma membrane, leading to cell death. In the absence of the bacteriocins encoded by the *blpMNO* genes, a 6A strain of pneumococcus was unable to compete with a serotype 4 strain (TIGR4) during nasopharyngeal colonisation [90]. Closely related commensal streptococci, including *Streptococcus oralis* and *Streptococcus mitis* also produce bacteriocins with anti-pneumococcal activity. These species may also compete with pneumococci for scarce metabolic resources in the nasopharynx, as suggested by recent genomic analyses [91]. A number of established epidemiological patterns provide the basis for our understanding of interspecies competition in the nasopharynx [92]. Pneumococcal colonisation has been consistently shown to be negatively associated with *Staphylococcus aureus* colonisation [93,94]. This pattern is replicated in populations around the world but is strikingly absent from people with HIV, indicating that competition between these bacteria might be mediated by the adaptive immune system [95,96]. In support of this, studies using co-colonisation of *S. aureus* and *S. pneumoniae* in animal models have shown that cross-reactive antibodies to putative dehydrogenases drive the competitive interaction, leading to reduced levels of *S. aureus* colonisation [97].

Multiple studies have revealed a positive association between colonisation by the pneumococcus and *Moraxella catarrhalis* [93,98,99]. However, when dissected experimentally, a complicated relationship between these organisms is revealed with the pneumococcus inhibiting *M. catarrhalis* in coculture [100]. A further well-studied interaction occurs between *Haemophilus influenzae* and the pneumococcus. Whether the interaction between these organisms is cooperative or competitive, though, again seems to depend on context. For example, observational studies in humans have indicated that in children prone to acute otitis media, levels of pneumococcal colonisation were negatively associated with non-typable *H. influenzae*, whereas in healthy children, colonisation by these two bacteria was positively associated [99]. Thus, the inflammatory environment of the upper airway during co-colonisation likely determines the relationship between *H. influenzae* and the pneumococcus. Mechanistic studies have revealed that peptidoglycan from *H. influenzae* activates the Nod1 pattern recognition receptor which promotes clearance of the pneumococcus from the upper airway via the activity of neutrophils [101]. A recent study demonstrated that ribosomal proteins of *Klebsiella pneumoniae,* another upper airway resident, contain peptide sequences specific for the pneumococcal Ami-AliA/AliB oligopeptide permease ABC transporter. These peptides are secreted by *K. pneumoniae* and suppress pneumococcal growth [102]. Collectively, these studies suggest a complex set of demands on the pneumococcus during colonisation, with inter- and intraspecies competition taking place against a backdrop of nutrient scarcity and a hostile host inflammatory response.

## Conclusion

Despite our increasingly sophisticated knowledge of pneumococcal biology, the tactics we use to prevent acute disease are still based on approaches pioneered decades ago. To fully understand the pneumococcus and devise new strategies to prevent infection, we need a deeper understanding of colonisation in context of the broader microbial and immunological environment in the nasopharynx. Our current perspective on colonisation is skewed: while we possess detailed mechanistic insights into certain potentially pathogenic species, our understanding of the diverse array of microbes not typically associated with disease remains limited. By comparing how these benign species interact with the host, we can gain richer insights into the behaviours and strategies of disease-associated residents like the pneumococcus. For instance, the pneumococcus exploits a conserved inflammatory programme in the airway to facilitate transmission to new hosts. However, using this inflammatory strategy comes at a cost—potentially shortening the duration of colonisation of the current host, and even compromising host viability by promoting pneumococcal translocation into the bloodstream. How do benign nasopharyngeal microbes spread between hosts? Current understanding of this is poor, and largely restricted to descriptive surveys that reveal broad patterns, such as that people who are in close environmental proximity are likely to have similar airway microbiomes, indicating likely microbial transmission between airways [103]. Such studies do not reveal the mechanisms which facilitate transmission. Similarly, what tactics do benign airway microbes use to persist during colonisation? Are they completely quiescent or do they elicit an immune response too? If the latter, how does this immunological environment affect acquisition and colonisation by the pneumococcus? There are several studies indicating there is active engagement of the immune system by airway commensals, and these could likely influence pneumococcal colonisation. For example, upper airway commensals that are robust activators of Nod2 enhance lower airway defences against the pneumococcus via IL-17A and GM-CSF [87]. Other work has shown that upper airway commensals, again via IL-17A signalling, stimulate defences in the upper airway to limit *K. pneumoniae* colonisation at this site [104]. It is through encapsulation that *K. pneumoniae* manages to overcome these defences and successfully establish colonisation [104]. Both examples demonstrate that members of the airway microbiota engage the local immune system, and this is also likely to be important during pneumococcal colonisation. By gaining more comprehensive knowledge of the broader community of benign microbes in the nasopharynx, we may uncover what sets potential pathogens like the pneumococcus apart from other nasopharyngeal microbes.

Pneumococcal metabolism is well characterised, largely through classical genetic approaches that disrupt individual pathways to reveal key factors supporting growth under tightly controlled laboratory conditions. What is missing, however, is understanding of how different metabolic pathways are coordinated over the course of a single carriage episode and in the transitions to disease or onward transmission. It is unlikely that pneumococci rely on a single carbon source for the duration of colonisation, but we currently lack the necessary understanding to predict how the pneumococcus selects which metabolic strategies to employ, and when to do so, although it is apparent that the interlinked Rgg/SHP and Tpr/Phr cell-cell communications systems are involved, given their sensitivity to nutritional cues and control of pneumococcal physiology [105–107]. Similarly, the metabolic resources accessible to pneumococci—and the immunological environments they encounter—likely vary significantly between individual human hosts, and this will be influenced by the microbiome and by direct effects of pneumococcal colonisation on host metabolism. Recent studies have highlighted the ability of pneumococci to influence BCAA availability in the nasopharynx [58] and to promote metabolic shifts in respiratory epithelial cell mitochondria that support bacterial survival [108]. Emerging approaches such as metabolomics, particularly when combined with human challenge models, offer powerful tools for achieving a systems-level understanding of pneumococcal biology in the human host. These approaches provide insight into its metabolic plasticity, ecological integration within the nasopharynx, and strategic exploitation of host immunity during transmission. Together, these interconnected factors underpin the success of the pneumococcus, and a deeper understanding of them could reveal new strategies to combat pneumococcal disease.
